# A Case Study Using Accelerometers to Identify Illness in Ewes following Unintentional Exposure to Mold-Contaminated Feed

**DOI:** 10.3390/ani12030266

**Published:** 2022-01-21

**Authors:** Sara C. Gurule, Victor V. Flores, Kylee K. Forrest, Craig A. Gifford, John C. Wenzel, Colin T. Tobin, Derek W. Bailey, Jennifer A. Hernandez Gifford

**Affiliations:** 1 Department of Animal and Range Sciences, New Mexico State University, Las Cruces, NM 88003, USA; gurules@nmsu.edu (S.C.G.); vvf@nmsu.edu (V.V.F.); kylee28@nmsu.edu (K.K.F.); colin.tobin@ndsu.edu (C.T.T.); dwbailey@nmsu.edu (D.W.B.); 2 Extension Animal Sciences and Natural Resources, New Mexico State University, Las Cruces, NM 88003, USA; cgifford@nmsu.edu (C.A.G.); jwenzel@nmsu.edu (J.C.W.)

**Keywords:** accelerometer, behavior, health, mold, mycotoxin, sheep

## Abstract

**Simple Summary:**

Observing the health and wellness of livestock is time consuming and costly. Sensor technologies can identify changes in animal activity, providing the potential to remotely monitor livestock health status and welfare. As part of another study, 10 ewes in a pen setting were monitored with near real-time accelerometers manufactured by Herddogg. Ewes were inadvertently fed moldy corn silage. The moldy feed was removed the following day and ewes displaying symptoms, such as reduced intake and difficulty walking, were treated under the direction of a veterinarian. Accelerometers showed a distinct decrease in activity for 4 days after the ewes were exposed to moldy feed. Accelerometers also showed an increase in activity of symptomatic ewes after treatment. Real-time and near real-time accelerometers have the potential to remotely detect changes in sheep activity that occur when animals become ill from mold contaminated feed and perhaps other illnesses, which could help producers monitor livestock health and provide a more timely response when they become ill.

**Abstract:**

Sensor technologies can identify modified animal activity indicating changes in health status. This study investigated sheep behavior before and after illness caused by mold-contaminated feed using tri-axial accelerometers. Ten ewes were fitted with HerdDogg biometric accelerometers. Five ewes were concurrently fitted with Axivity AX3 accelerometers. The flock was exposed to mold-contaminated feed following an unexpected ration change, and observed symptomatic ewes were treated with a veterinarian-directed protocol. Accelerometer data were evaluated 4 days before exposure (d −4 to −1); the day of ration change (d 0); and 4 days post exposure (d 1 to 4). Herddogg activity index correlated to the variability of minimum and standard deviation of motion intensity monitored by the Axivity accelerometer. Herddogg activity index was lower (*p* < 0.05) during the mornings (0800 to 1100 h) of days 2 to 4 and the evening of day 1 than days −4 to 0. Symptomatic ewes had lower activity levels in the morning and higher levels at night. After accounting for symptoms, activity levels during days 1 to 4 were lower (*p* < 0.05) than days −4 to 0 the morning after exposure. Results suggest real-time or near-real time accelerometers have potential to detect illness in ewes.

## 1. Introduction

Behavior is a basic indicator of an animal’s health and wellness state [1], which highlights the importance of knowing normal behavior patterns of an individual animal. Producers observe animal behavior to assess health status and make management decisions, in an effort to maintain animal welfare and increase productivity of their operation. Animals experiencing illness or painful conditions typically demonstrate changes in appearance, appetite, posture, and behavioral patterns [2,3]. However, prey animals like sheep tolerate pain and injuries without displaying an overt change in behavior as a means to limit vulnerability towards predators and, subsequently, increase their chances of survival [4,5]. The ability of sheep to mask their pain by maintaining a stoic demeanor may hinder early detection of subtle behavior changes. Behavioral irregularities related to painful events can be difficult to identify by observation alone in an intensive system, where space is limited, and an animal can blend within a group of animals. Monitoring livestock may also be difficult in a pasture setting due to low frequency of human observation. Human observation of livestock can be labor intensive; however, it is crucial to detect subtle changes in behavior that might be associated with illness to allow prompt treatment before an animal’s health is further compromised.

A great deal of recent research has focused on remote monitoring of livestock. Most of the research has focused on on-animal sensors such as global position system (GPS) tracking and accelerometers [6]. These systems can be used to identify changes in normal activity patterns of livestock, which can be an indication of illness or other animal well-being concerns [7]. Tracking has been used successfully to detect parturition in sheep [8,9] and simulated water failure in cattle [10]. However, value of GPS tracking is limited in intensive and pen situations because the spatial movements of livestock are constrained.

The application of accelerometers has been utilized to across livestock systems to identify normal behavior and changes due to shifts in well-being and health status. Accelerometers have been used to detect parturition in sheep [11,12]. Accelerometers have also been used to monitor rumination [13], grazing activity [14], and drinking behavior [15] in cattle. Multiple studies have utilized accelerometers and have shown reductions in activity and movement intensities. Tobin et al. [16] used accelerometers to detect the reduction of movement intensity due to bovine ephemeral fever in heifers. Ikurior et al. [17] deployed tri-axial accelerometers to detect changes in lamb activity due to gastrointestinal nematode infection.

For intensive operations, more options for remote monitoring are available than for rangeland and pasture-based systems [7]. Thermal imaging has potential to remotely monitor livestock health and well-being [18]. Rumen boluses can also be used to remotely monitor changes in body temperatures of cattle [19]. Indwelling vaginal temperature sensors can be utilized to determine internal temperatures [20]. The usage of multiple sensors is to provide automatic, continuous monitoring of individual animals [21].

Deviation from normal activities can also be a consequence of an abrupt change in diet. Exposure of livestock to toxins present in feed can cause immediate and lethal consequences that require prompt intervention. Molds and mycotoxins are common contaminants in feedstuffs, which are most frequently observed in hay and silages [22]. Mycotoxins impact on animal health is based on a number of factors including level of exposure to feed contamination and animal sensitivity due to immunosuppression [23]. Differentiating molds and mycotoxins’ effects on animal health and performance are challenging [24], as molds may be present without producing toxins [25]. Mycotoxins are a large and complex group of secondary metabolites, which are produced by fungi and certain varieties of molds that stimulate a multitude of toxic responses in humans and animals [22,23]. These low-molecular-weight metabolites are toxic when consumed even in low concentrations [25]. Mycotoxicosis from consumption of feedstuffs containing toxic metabolites typically comes about without producer awareness and transpires over a long period of exposure [22]. Exposure to mycotoxins can also have escalating and undetectable consequences on animal health in early stages [22]. Reduced feed intake and prolonged duration of feeding intervals may occur after exposure to moldy feed due to decreased feed digestibility [26]. Numerous mycotoxins are able to modify the rumen microflora, resulting in decreased milk production, mild diarrhea, and poor feed conversion [26].

Low levels of chronic exposure to mycotoxins are not always noticed and behavioral change may be challenging to detect due to the rumen’s ability to degrade, deactivate, and bind toxic molecules [22]. However, detecting behavioral changes at the onset of those animals exhibiting acute mycotoxicosis, where detrimental signs of disease are present, is critical to allow the manager to respond before health is too heavily altered. Sensor technologies possess the ability to detect minute changes in activity, to aid in monitoring livestock. Studies suggest sensor technologies are capable of detecting a variety of abnormalities in behavior linked to changes in animal health [16]. The aim of this case study was to investigate the potential to remotely monitor changes in behavior associated with illness caused by mold-contaminated feed.

## 2. Materials and Methods

### 2.1. Site and Animals

All procedures were approved by the New Mexico State University Animal Care and Use Committee (2019-007). This analysis was based on a separate study evaluating parturition and was conducted on the campus of New Mexico State University at the West Sheep Unit research facility. Twenty-five fine wool Debouillet, ewes averaged 3 years (± 0.2 SEM) of age and weighed 79 kg (±3.2) at the start of the parturition study. All animals were observed daily and were healthy at the onset of this study. Ewes were housed in a single pen (18.3 m × 9.1 m) to evaluate the potential of accelerometers to detect parturition-related behavior events [11]. Each ewe was fed 1.8 kg of chopped alfalfa in the morning (0800 h) and supplemented with 0.22 kg of cracked corn both morning (0800 h) and afternoon (1600 h). An unexpected ration change to corn silage occurred on 16 April 2019. Ewes were either in late gestation or in the first 21 days of lactation at the time of the unexpected feed change (16 April 2019) described below. Throughout the trial ewes had ad libitum access to water, mineral, and salt.

### 2.2. Accelerometers

A tri-axial Axivity AX3 MEMS accelerometer (Axivity Ltd., Newcastle, UK) was attached to an Allflex ear tag (Allflex USA Inc., Dallas, TX, USA) with shrink wrap tubing, then randomly placed in either the left or right ear of 13 ewes prior to parturition. Accelerometer ear tags were placed on ewes on 13 March 2019 and were removed on 12 May 2019. Accelerometers were charged prior to deployment to last a duration minimum of 30 days (study duration). Axivity accelerometers were configured to collect acceleration signals at a sample rate of 12.5 Hz measuring longitudinal movements of the horizontal *x*-axis (left and right), longitudinal *y*-axis (forward and backward), and vertical *z*-axis (up and down). The dimensions of each accelerometer were 23 mm × 32.5 mm × 7.6 mm and weighed 11 g. Accelerometer movements were subsequently stored on the NAND Memory within the device. Accelerometers were removed post-study to retrieve data via USB connection to the OmGui Axivity computer software. The OmGui program downloads data from the accelerometer, allows for manipulation for desired study period, and stores raw data as a .CWA file, not compatible with Microsoft Excel (Microsoft Corporation, Redmond, WA, USA).

A HerdDogg biometric accelerometer ear tag (HerdDogg, Inc., Ashland, OR, USA), was attached to 25 ewes prior to parturition. On the 13 ewes that received an Axivity AX3 accelerometer, a HerdDogg accelerometer was attached on the opposing side ear. HerdDogg accelerometers were configured to collect tri-axial acceleration signals at a sample rate of 24 Hz. The accelerometer signals from the X, Y and Z axes were processed in the HerdDogg tag and condensed into one proprietary index value every 6 min. The raw X, Y, and Z data is not stored on the tag and is only used to calculate the index value. Ambient temperature and temperature measured by a sensor next to the ear were also transmitted in 6 min epochs. Data transmitted from the ear tag was gathered by HerdDogg’s “DoggBone” receiver, which transmits the data via cellular network technologies to a website and smart phone app where it can be viewed. The dimensions of the HerdDogg ear tag were 76.2 mm × 38.1 mm × 12.7 mm and weighed 25 g.

### 2.3. Daily Animal Observation Protocol

Ewes from the West Sheep Unit research facility were regularly checked at 0800 h at time of feeding and 1300 h daily for any signs of abnormal behavior or illness within the animals. However, when symptoms were first presented, ewes were checked for change in behavior and locomotion four times daily. Symptomatic ewes were then treated with a protocol directed by a veterinarian (discussed below).

### 2.4. Ration Change

Ewe’s ration was unexpectedly shifted overnight on 16 April 2019 from an alfalfa-corn mixture to a corn-silage. No observable presence of mold or other contaminants were noted upon feeding the corn-silage. The entire flock from the West Sheep Unit were exposed to the corn-silage feed, which was later determined to have been contaminated with mold, including the ewes with a HerdDogg ear tag and Axivity AX3 accelerometer. The unintentional exposure of the ewes to the contaminated feed occurred during late pregnancy or early lactation. Contaminated feed was removed from feed bunks the following day (17 April 2019) in the afternoon after overt detrimental changes to health status were observed. A feed sample was collected for feed analysis and sent to be performed by SDK Laboratories (SDK Laboratories Inc., Hutchinson, KS, USA) (Table 1). Ration was switched to 100% alfalfa on 18 April 2019, and ewes were treated based on the protocol below. Animals were moved to a separate pen for treatment.

### 2.5. Symptoms and Resulting Treatments

**Figure 1:**
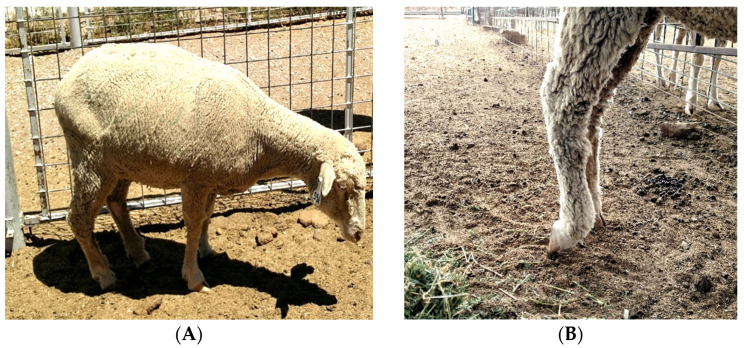
Predominant symptoms observed included: (**A**) ewe demonstrating difficulty walking and standing with its head down; (**B**) knuckling of the feet as a result of ingested contaminated-feed.

Within 24 h of exposure; one ewe demonstrated difficulty standing, kept her head down and ultimately died within the first day after exposure (this ewe was not included in the current study). Within two days after exposure, seven of the 25 ewes in the pen discontinued eating and had difficulty walking. Three days post-exposure, five ewes persisted with symptoms of difficultly walking and not eating, even after treatment. Four days after exposure, all but one symptomatic ewe returned to a normal state. Symptoms recorded included: no feed intake, difficulty or weakness in walking, head held down, knuckling of the feet (Figure 1), and continuous shifting of body weight from one leg to another.

Treatments began on the morning of 18 April 2019. All ewes showing symptoms were treated with 60 mL of sodium bicarbonate, 10 mL of Bismuth subsalicylate, 3 mL of Banamine^®^, and were placed into a separate sick pen.

### 2.6. Data Collected and Evaluated

All ewes in the flock were evaluated opportunistically after unexpected exposure to mold contaminated corn silage. Observation data were recorded for ewes exhibiting abnormal behavior each day after feed exposure until symptoms subsided. Daily treatments were also recorded for each ewe.

The unexpected feeding of moldy feed occurred near the end of the expected battery life for the Axivity accelerometer batter life (30 days). Only five ewes had nearly complete data (>95% of expected data recordings) during this study period. The Herddogg accelerometer tags were an earlier version of the technology and sometimes did not transfer all the index values to the Dogbone receiver. Fifteen of the 25 ewes with HerdDogg ear tags could not be used in the study due to inconsistent missing index values within the data sets.

Accelerometer data recorded by HerdDogg ear tags were used to evaluate ewe behavior 4 d prior to exposure to the moldy corn silage (days −4 to −1), the day moldy corn silage was first introduced (day 0), and 4 d post-exposure (days 1 to 4). This data set allowed us to evaluate ewe behavior in this case study using a before and after analysis. Ten ewes had complete HerdDogg accelerometer data during this 9 d period and were included in the study. Three of these 10 ewes displayed symptoms and were treated. HerdDogg accelerometer ear tags provide an index value every 6 min that the manufacturer states is related to animal activity. These index values were averaged each hour for statistical analyses.

To gain a better appreciation of the proprietary HerdDogg accelerometer index, the correlation between the accelerometer index and metrics calculated from the Axivity accelerometer were calculated. Five ewes had both Axivity and Herddogg data for 8 days at the time of the study (12 April to 20 April). Accelerometer data were retrieved using the Axivity proprietary software Version 1.0.0.37, OmGui, and condensed into 10 s epochs using Anaconda Python programming. Movement intensity (MI) and signal magnitude area (SMA) was calculated for accelerometer reading.

(1) $$\mathrm{MI}=\frac{1}{T}\overset{T}{\underset{t=1}{\sum }}\sqrt{\left(Ax^{2}\right)+\left(Ay^{2}\right)+\left(Az^{2}\right)}\left(t\right)$$

(2) $$\mathrm{SMA}=\frac{1}{T}(\sum_{t=1}^{T}\left|Ax\left(t\right)\right|+\sum_{t=1}^{T}\left|Ay\left(t\right)\right|+\sum_{t=1}^{T}\left|Az\left(t\right)\right|)$$

where **Ax**, **Ay**, and **Az** are the Axivity accelerometer readings from the *x*, *y*, and *z* axes, respectively as described in detail by Gurule et al. [11] and Tobin et al. [16].

The mean MI, range of MI, standard deviation of MI, mean of signal magnitude area (SMA), mean of the x axis, mean of the y axis, and mean of the z axis were calculated for each 10 s epoch. These metrics calculated from the Axivity data were averaged by hour and paired with corresponding hourly averages of the HerdDogg accelerometer index for correlation analyses. We paired Axivity metrics and the HerdDogg index data (by hour each day) for each ewe and pooled the data from all ewes. We used MI as a metric for the Axivity data, because the Herddogg manufacturer reported to us that their proprietary index was a compilation of data from all 3 axes and was similar but not the same as MI [27].

The placement of the Axivity and HerdDogg accelerometer in the left or right ear could potentially affect the values accelerometer readings. However, there were only 5 ewes with complete data so there was not sufficient data to compare the effect of placing the tag in the left or right ear. In addition, we used a before and after analysis of individual ewes so the impact of placement of the tag was accounted for by the subject of the repeated measures model (see below).

### 2.7. Statistical Analyses

The hourly averages of the HerdDogg accelerometer index were classified into four periods, morning (0800 to 1100 h), midday (1100 to 1700), evening (1700 to 2000), and night (2000 to 0800). Due to symptoms including reduced feed intake and difficulty walking, periods were classified into periods where ewes typically had different activity levels based on visual observations. Morning was the period when ewes were fed and were typically active. Midday had lower activity than feeding, activity often increased during the evening, and night had the lowest activity level. These diurnal patterns in sheep activity are typical of domestic sheep [28] had to be accounted for in the analyses in order to accurately compare before and after the moldy forage was fed.

The HerdDogg accelerometer index data were analyzed using repeated measures in PROC MIXED of SAS [29]. The fixed effects were day (−4 to 4), period (morning, midday, evening, and night), hour within period, and the period × day interaction. Day 0 was the day ewes were first exposed to the moldy feed. Days −4 to −1 were the four days prior to feeding the moldy corn silage, with day −1 being the day immediately prior to feeding. Days 1 to 4 were the days following the exposure to the moldy feed, with day 1 being the day that moldy corn silage was removed from the feed bunks midday, and day 2 being the day treatment for the symptomatic ewes began following the veterinarian directed protocol discussed above. The subject of the repeated measures model was ewe. The covariance structures evaluated were compound symmetry, autoregressive order 1, and unstructured [29]. The structure used of the three was based on the lowest Akaike Information Criterion (AIC). Pairwise comparisons of days, periods, and the day × period interactions were evaluated using the PDIFF function of PROC MIXED.

A second similar repeated measures analysis was completed with the addition of the fixed effect of symptom presence. Specifically, the model included day, period, hour within period, and symptom presence. The two-way interactions and three-way interactions of these fixed effects were also evaluated. Ewe was the subject and the covariance structure with the lowest AIC was autoregressive order 1. A three-way interaction of day, period, and symptom presence was not detected (*p* = 0.49), so it was dropped from the model.

Simple (Pearson) correlation coefficients were calculated between the HerdDogg accelerometer index and the metrics calculated from the Axivity accelerometers. Pairs of the HerdDogg index and Axivity metrics were based on hourly measures collected over 8 days (see above). Separate correlation coefficients were calculated for each of the five ewes with complete data during this period. In addition, correlation coefficients were collected for the pooled data of all five ewes.

The HerdDogg accelerometer index was used to evaluate the changes in behavior before and after feeding the moldy corn silage, due to more ewes being monitored with HerdDogg during the period that the silage was fed (10 versus five). Only one of the ewes monitored with Axivity accelerometers showed any symptoms versus three ewes with HerdDogg. Axivity data was not complete for the 9-day study (days −4 to 4) for the majority of the five ewes due to loss of battery charge in the Axivity accelerometer. In addition, the HerdDogg accelerometer tags are commercially available and transmit to a website and smart phone app in real time. Axivity accelerometers are “store on board” and accelerometers must be removed from the ewe to download data and are not applicable to commercial livestock operations but serve as a valuable research tool.

## 3. Results

### 3.1. Evaluation without Considering Symptom Presence

**Figure 2:**
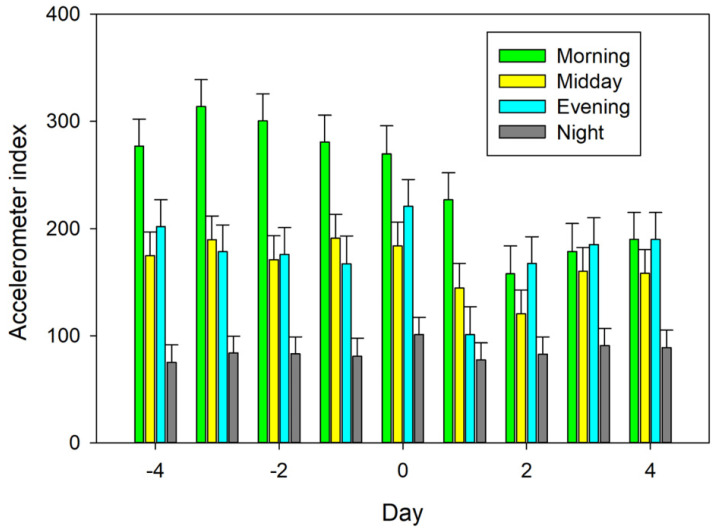
HerdDogg accelerometer index means of periods, morning (0800 to 1100 h), midday (1100 to 1700 h), evening (1700 to 2000 h), and night (2000 to 0800). Sheep were fed at 0800 h. Ten ewes were monitored for 4 d (−4 to −1) prior to receiving an unexpected ration change of moldy corn silage on days 0 and 1. Moldy feed was removed in the evening of day 1 when symptoms were first observed. Error bars represent standard errors.

No differences in the Herddogg activity index among days (−4 to 4) were detected (*p* = 0.16). Activity varied (*p* < 0.001) among periods (Figure 2). Morning had greater (*p* < 0.001) activity than midday, evening, or night, and night had lower (*p* < 0.0001) activity than morning, midday, and evening. No differences (*p* = 0.18) were detected between evening and midday. Activity also varied (*p* < 0.001) among hours within period.

There was an interaction (*p* < 0.001) between day and period (Figure 2). No differences in activity were detected (*p* > 0.01) in the morning between days −4 to 0. Activity in the morning was lower (*p* < 0.05) on days 1 to 4 than days −4 to −2. No differences in morning activity were detected (*p* > 0.05) between day −1 and days 0 and 1. Activity in the morning was lower (*p* ≤ 0.01) on days 2 to 4 than day −1. Activity in the morning on days −1 and 0 were higher (*p* < 0.01) than days 2 to 4. Morning activity on day 2 was lower (*p* = 0.04) than day 1. No differences (*p* > 0.05) were detected between morning activity on day 1 than days 3 and 4. No differences in morning activity were detected (*p* > 0.05) among days 2, 3, and 4.

No differences among days in activity at midday were detected (*p* > 0.05). Activity in the evening was lower (*p* < 0.05) on day 1 than days −4, −3, −2, 0, 2, 3, and 4. No differences in activity were detected (*p* = 0.14) between day 1 and day −1 in the evening. No other differences in evening activity were detected (*p* > 0.05) among the other days. No differences in nighttime activity were detected (*p* > 0.05) among days.

### 3.2. Evaluation Considering Symptom Presence

Similar to the previous analysis, the Herddogg activity index varied among days (*p* = 0.03). As expected, no differences in activity were detected (*p* > 0.05) prior to feeding the moldy corn silage (days −4 to day −1). No differences in activity were detected (*p* > 0.05) between day 0 and days −4 to day −1. Activity was lower (*p* < 0.05) on days 1 and 2 than days −4 to 0. No differences in activity were detected (*p* > 0.05) between days 3 and 4 and days −4 to 0. No differences were detected (*p* > 0.05) between day 1 and 2 and days 3 and 4.

Activity differed (*p* < 0.001) among periods with morning having greater (*p* < 0.001) activity than midday, evening or night, and night being lower (*p* < 0.0001) than morning, midday, and evening. No differences (*p* = 0.36) were detected between evening and midday. Activity also varied (*p* < 0.001) among hours within period.

**Figure 3:**
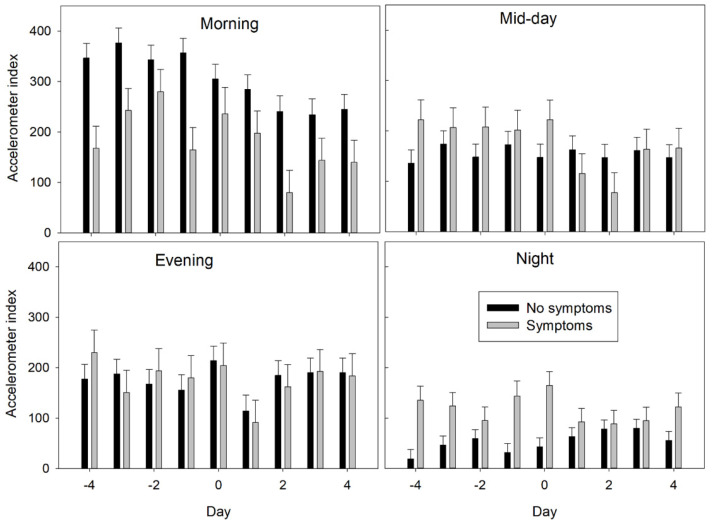
HerdDogg accelerometer index values for ewes demonstrating symptoms (e.g., reduced feed intake and difficulty walking) during the morning (0800 to 1100 h), midday (1100 to 1700 h), evening (1700 to 2000 h), and night (2000 to 0800 h). Sheep were fed at 0800 h. Ten ewes were monitored for 4 days (−4 to −1) prior to receiving an unexpected ration change of moldy corn silage on days 0 and 1. Moldy feed was removed in the evening of day 1 when symptoms were first observed. Error bars represent standard errors.

No differences in activity were detected (*p* = 0.47) between ewes displaying symptoms and ewes not displaying symptoms. However, there was an interaction (*p* < 0.001) between period and presence of symptoms (Figure 3). Ewes displaying symptoms had lower (*p* < 0.001) activity during the morning and higher (*p* < 0.001) activity at night than ewes that did not display symptoms. No differences in activity were detected (*p* > 0.05) between ewes displaying symptoms and ewes not displaying symptoms during midday and evening periods.

**Figure 4:**
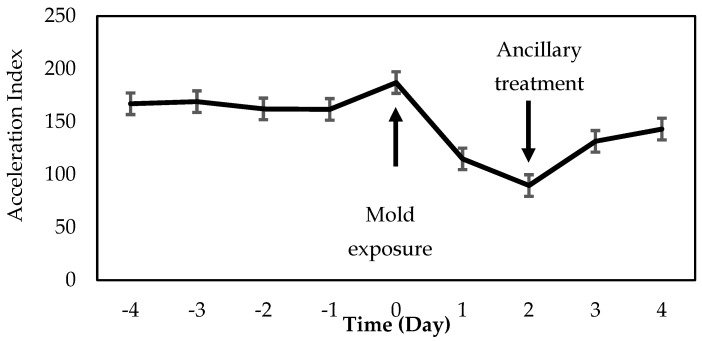
Mean daily accelerometer readings from the HerdDogg accelerometer over a 9-d period, before and after mold contaminated feed was fed to ewes. Day 0 was the day ewes were exposed to the feed (indicated by arrow) and day 2 was the day treatment for symptomatic ewes began, following a veterinarian directed protocol (indicated by arrow). Error bars represent standard errors.

Herddogg accelerometer data from the three ewes displaying symptoms identified a decrease in activity (*p* = 0.03) for 2 days after feed exposure compared to the 4 days before exposure (Figure 4). Three days after exposure and 2 days after treatment, no difference in activity was detected (*p* > 0.05) between pre- and post-mold exposure levels.

### 3.3. Correlations between HerdDogg and Axivity Metrics

Correlations between the HerdDogg accelerometer index and Axivity accelerometer metrics were not consistent across the five ewes evaluated (Table 2). Across all ewes, the HerdDogg accelerometer index was most correlated to the standard deviation of MI (0.62) and the minimum of MI (−0.65). For all but one of the five ewes, there was a strong correlation (>0.60) between MI standard deviation and the HerdDogg index (Table 2). For ewes 601 and 742, mean MI was only weakly correlated to the HerdDogg accelerometer index. Stronger correlations were found between the minimum and standard deviation of MI for ewe 742 (Table 2). The correlations between the HerdDogg index and the Axivity metrics were weak for ewe 601. Mean SMA was weakly or moderately related to the HerdDogg index. The HerdDogg accelerometer index was weakly correlated to the means of the x, y, and z axes of the Axivity accelerometer (Table 2).

## 4. Discussion

The HerdDogg accelerometer index is designed to monitor livestock activity (https://herddogg.com accessed on 15 June 2021). Metrics from the Axivity accelerometers were more correlated to the mean and variation (minimum and standard deviation) of MI than the means of individual axes and SMA. Results from the associated study, Gurule et al. [12], showed that greater MI values and greater variation in MI were associated with active behavior. Similarly, Fogarty et al. [30] found that ewe activity was most associated with the variation in accelerometer metrics rather than the means of the metrics. Results from the present study, suggest that the HerdDogg accelerometer tags provide an index that reflects the variation in accelerometer movements Changes in the HerdDogg accelerometer index are related to the variation in the head movements of the ewe. More variation in head movements likely means higher HerdDogg index values and likely more ewe activity.

When all ewes were evaluated without considering if the ewe displayed symptoms, no differences in activity when considering the entire day were detected. However, there was a clear identification of a reduction in activity during the morning after ewes were fed. Decrease in activity monitored by the HerdDogg ear tag showed a clear decrease from 0800 to 1100 h after the moldy corn silage was fed. There was also a decrease in activity in the evening (1700 to 2000 h), when ewes began displaying symptoms and the moldy feed was removed from the bunk. This HerdDogg monitoring data was transmitted and recorded using their website. This change in behavior could have alerted the caretakers, had an algorithm been developed to detect the change in morning behavior, most likely feeding behavior. Results from this study show the potential to detect a decrease in feeding behavior. The accelerometer index dropped to less than half of its previous values (days −4 to 0) on day 2 (Figure 4). This decrease in activity was also apparent in the HerdDogg accelerometer reading during the evening of day 1 when symptoms were first observed.

When ewes that displayed symptoms were included in the statistical model, there was a clear change in activity among days. Activity on days 1 and 2 were lower (after feeding) than previous days (days −4 to 0). After treatment on days 3 and 4, no differences in activity could be detected from pre-mold exposure (days −4 to −1). Both ewes with symptoms and without systems displayed lower activity after feeding moldy feed, but the change in behavior of ewes without symptoms was less than ewes with symptoms and limited to the morning period (Figure 4).

Ewes that displayed symptoms displayed different diurnal activity patterns from ewes that did not have symptoms. Ewes with symptoms had lower activity in the morning (normal feeding period) and greater activity at night than ewes that did not display symptoms. These behavioral differences may or may not be associated with the susceptibility of the ewes to mycotoxins. Accounting for the differences in activity of the ewes displaying symptoms did improve the precision of the statistical model (lower AIC value) and allow us to detect a difference among days. More research is needed to determine if diurnal activity patterns affect the susceptibility of ewes to mycotoxins.

The decrease in activity that was detected after moldy forage was fed to the ewes could occur if ewes became ill from other sources. For examples, Tobin et al. [16] found that the activity of heifers diagnosed with bovine ephemeral fever declined. Much more research is needed before the exact cause of illness can be determined from accelerometers and other on-animal sensors. However, this does not negate the value remote monitoring with on-animal sensors. If the system identifies a potential problem as indicated by a deviation from an animal’s normal behavior and notifies the caretakers, the staff can locate and observe the animal in question and make diagnosis. Providing prompt notice of potential health concerns from a change from normal behavior should help caretakers quickly respond and provide treatment if needed.

In this study, the majority of the HerdDogg accelerometer ear tags were not able to successfully transfer all the data from the tag to the Dogbone reader. However, the technology in this system continues to be developed and improved. The problems transferring data in this study likely would not occur with current versions of the HerdDogg tags and Dogbones. The distance that data can be transmitted has increased from approximately 10 m to 100 m [31].

Mycotoxins may not always be present in moldy feed; nonetheless, mold itself can cause negative effects on health and production [32]. It has been suggested that ruminants are less susceptible to mycotoxins by conversion in the flora to biologically inactive metabolites; however, that does not apply to all mycotoxins that may contaminate feed [33]. In a study by Kiessling et al. [34], mycotoxins were incubated in rumens of sheep and cattle, mycotoxin concentration was measured and demonstrated that aflatoxin B_1_ and deoxynivalenol were not degraded by rumen microorganisms. A common practice in ill ruminants is transfaunating the rumen, by providing microorganisms from a healthy donor to re-establish the microbial population in a sick animal [35]. One ewe exposed to the moldy silage had persisting symptoms (six days post-exposure) even after treatment, so a rumen transfaunation was performed using a healthy donor ewe.

There are over 300 known mycotoxins [25], which can make detection difficult, as only three to four of the most common mycotoxins are tested [32]. Not all mycotoxins can be detected and conjugated mycotoxins can be masked in routine testing by commercial laboratory analysis [19,36]. In addition, detection can be compromised by the variance in a representative sample, as molds can produce large quantities of mycotoxins in small areas and are not evenly distributed in the feedstuffs [36]. Although no mycotoxins were detected in our study, high mold count has been demonstrated to produce potent mycotoxins effecting animal health [36]. Table 1 of the present study demonstrates no detectable mycotoxin. However, a negative test of mycotoxin with animals showing symptoms suggests mycotoxin was present but not detectable [32].

Stage of production may be an important factor in resulting symptoms of mycotoxin exposure. Animals experiencing stressful situations, such as parturition, may have more pronounced symptoms due to their already suppressed immune system [36]. Applebaum et al. [37] observed that cows that were treated with impure aflatoxin B_1_ demonstrated a significant decrease in milk production. During the lambing season following the exposure to mold-contaminated feed, ewes from our study displayed low milk production, as numerous lambs were being supplemented with synthetic milk.

Animals may respond to mold and mycotoxin exposure differently based on duration and dose of exposure, stress, and age [38]. One of the most prominent symptoms of mycotoxicosis is reduced feed intake or feed refusal [39] and knuckling of the feet (Figure 1). Ewes in the present study that displayed acute toxicosis refused feed, which could be detected in accelerometer data when compared to normal diurnal activity patterns. These sensor technologies are fixed on a lightweight ear tag providing minimal to no obstruction, allowing natural movement of the animal. Therefore, these sensor technologies have potential to discriminate between subtle changes in normal behavior, indicating deviation in animal’s health status before observable health consequences are notable. This may be beneficial to the manager by minimizing their economic losses and indicating change in activity before complications arise.

## 5. Conclusions

HerdDogg accelerometer ear tags were capable of detecting changes in activity and behavior of ewes that were exposed to moldy feed in this case study. Several ewes displayed symptoms, such as reduced or no feed intake and difficulty walking. This ear tag monitoring system is commercially available and transmits the data in real time in a pen setting. Continued developments in remote monitoring systems will facilitate reliable transmission of data from on-animal sensors to the manager. Development of algorithms that can detect changes in behavior and activity could be used to alert managers when ewes in a pen setting face a well-being concern such as consumption of mycotoxins in moldy feed. More research is needed for development of such algorithms and validation of this “real-time” technology for remotely monitoring sheep well-being in a pen setting.

## Figures and Tables

**Table 1:** Feed analysis performed by SDK Laboratories. SDK Laboratories NOTE: Should discontinue feeding due to high mold count or dilute 1:3 with “clean feed” and feed only to non-pregnant animals or animals not under stress.

Parameter	Dry Basis	As Received	Unit
Moisture		43.12	%
Dry Matter		56.88	%
Protein, Crude	7.17	4.08	%
ADF-Acid Detergent Fiber	19.60	11.15	%
aNDF—Neutral Detergent Fiber	30.66	17.44	Mcal/lb
NEL: Net Energy-Lactation	0.79	0.45	Mcal/lb
NEG: Net Energy-Gain	0.54	0.31	Mcal/lb
NEM: Net Energy-Maintenance	0.87	0.49	%
TDN: Total Digestible Nutrients	75.75	43.09	%
Calcium	0.31	0.18	%
Phosphorus	0.23	0.13	%
Potassium	0.67	0.38	%
Magnesium	0.22	0.13	%
Aflatoxin		Less than 5	ppb
Fumonisin		4.3	ppm
Zearalenone		73.6	ppb
Vomitoxin		Less than 0.5	ppm
Mold		10,000,000	cfu/g
RFV-Related Feed Value	233		s.u.

**Table 2:** Correlation coefficients between the hourly average of the HerdDogg accelerometer index and hourly means of metrics calculated from an Axivity accelerometer attached to the opposite ear on the same ewe. Correlation coefficients were calculated from 8 d of data on five ewes, both individually and pooled.

Axivity Metric	Correlation Coefficient between HerdDogg Accelerometer Index and the Metric Calculated from the Axivity Accelerometers					
	Ewe 453	Ewe 544	Ewe 545	Ewe 601	Ewe 742	Pooled
*X*-axis Mean	−0.23	−0.29	0.18	0.00	0.39	0.16
*Y*-axis Mean	−0.07	−0.19	0.13	0.23	0.09	−0.03
*Z*-axis Mean	−0.10	−0.25	−0.40	0.04	0.31	0.21
MI Mean	0.62	0.52	0.66	0.21	0.10	−0.01
SMA Mean	0.37	0.40	0.51	0.09	0.58	0.21
MI Minimum	−0.76	−0.58	−0.63	−0.23	−0.77	−0.65
MI Standard Deviation	0.82	0.66	0.67	0.25	0.78	0.62

## Data Availability

The data presented in this study are available on request from the corresponding author.
